# Genetic characterization of 2008 reassortant influenza A virus (H5N1), Thailand

**DOI:** 10.1186/1743-422X-7-233

**Published:** 2010-09-16

**Authors:** Alongkorn Amonsin, Jiradej Lapkuntod, Kamol Suwannakarn, Pravina Kitikoon, Sanipa Suradhat, Rachod Tantilertcharoen, Supanat Boonyapisitsopa, Napawan Bunpapong, Manoosak Wongphatcharachai, Trong Wisedchanwet, Apiradee Theamboonlers, Yong Poovorawan, Jiroj Sasipreeyajan, Roongroje Thanawongnuwech

**Affiliations:** 1 Emerging and Re-emerging Infectious Diseases in Animals, Research Unit, Faculty of Veterinary Science, Chulalongkorn University, Bangkok, Thailand; 2 Department of Veterinary Public Health, Faculty of Veterinary Science, Chulalongkorn University, Bangkok, Thailand; 3 Department of Pathology, Faculty of Veterinary Science, Chulalongkorn University, Bangkok, Thailand; 4 Department of Medicine, Faculty of Veterinary Science, Chulalongkorn University, Bangkok, Thailand; 5 Department of Microbiology, Faculty of Veterinary Science, Chulalongkorn University, Bangkok, Thailand; 6 Center of Excellence in Clinical Virology, Faculty of Medicine, Chulalongkorn University Bangkok, Thailand

## Abstract

In January and November 2008, outbreaks of avian influenza have been reported in 4 provinces of Thailand. Eight Influenza A H5N1 viruses were recovered from these 2008 AI outbreaks and comprehensively characterized and analyzed for nucleotide identity, genetic relatedness, virulence determinants, and possible sites of reassortment. The results show that the 2008 H5N1 viruses displayed genetic drift characteristics (less than 3% genetic differences), as commonly found in influenza A viruses. Based on phylogenetic analysis, clade 1 viruses in Thailand were divided into 3 distinct branches (subclades 1, 1.1 and 1.2). Six out of 8 H5N1 isolates have been identified as reassorted H5N1 viruses, while other isolates belong to an original H5N1 clade. These viruses have undergone inter-lineage reassortment between subclades 1.1 and 1.2 and thus represent new reassorted 2008 H5N1 viruses. The reassorted viruses have acquired gene segments from H5N1, subclade 1.1 (PA, HA, NP and M) and subclade 1.2 (PB2, PB1, NA and NS) in Thailand. Bootscan analysis of concatenated whole genome sequences of the 2008 H5N1 viruses supported the reassortment sites between subclade 1.1 and 1.2 viruses. Based on estimating of the time of the most recent common ancestors of the 2008 H5N1 viruses, the potential point of genetic reassortment of the viruses could be traced back to 2006. Genetic analysis of the 2008 H5N1 viruses has shown that most virulence determinants in all 8 genes of the viruses have remained unchanged. In summary, two predominant H5N1 lineages were circulating in 2008. The original CUK2-like lineage mainly circulated in central Thailand and the reassorted lineage (subclades 1.1 and 1.2) predominantly circulated in lower-north Thailand. To prevent new reassortment, emphasis should be put on prevention of H5N1 viruses circulating in high risk areas. In addition, surveillance and whole genome sequencing of H5N1 viruses should be routinely performed for monitoring the genetic drift of the virus and new reassorted strains, especially in light of potential reassortment between avian and mammalian H5N1 viruses.

## Findings

**Figure 1:**
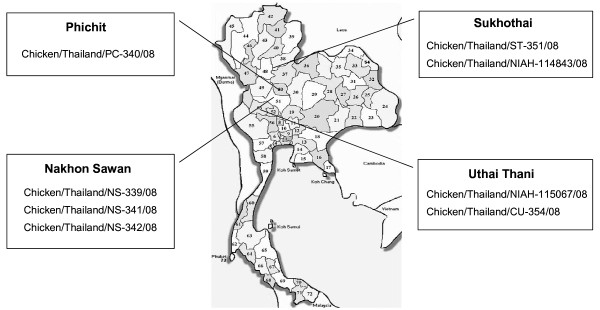
**Map of Thailand, provinces with 2008 H5N1 outbreaks are shown**.

H5N1 influenza A virus has caused avian influenza (AI) outbreaks worldwide. In Thailand, 7 major AI outbreaks have been reported since early 2004 [1-3]. In January 2008, outbreaks of H5N1 virus occurred in two provinces, Nakhon Sawan and Phichit. The outbreak in Nakhon Sawan affected 60,000 birds in a broiler farm and chicken in nearby backyards, while the outbreak in Phichit occurred among backyard chicken. In November 2008, H5N1 outbreaks were also reported in two provinces, Sukhothai and Uthai Thani. Both outbreaks occurred among backyard poultry in villages (Fig 1). Currently, at least two clades of influenza A virus (H5N1) have been reported in Thailand including clade1 viruses which are predominant in lower-north and central Thailand and clade2.3.4 viruses which are predominant in northeast Thailand [1,3,4]. Clade1 H5N1 viruses in Thailand have been further divided into 3 distinct subclades including the original clade1 (CUK2-like), clade1.p1 (PC168-like) and clade1.p2 (PC170-like) [3,5]. One study has documented evidence of genetic reassortment of H5N1 viruses in Thailand in 2007 [6]. In this study, we have comprehensively characterized the 2008 H5N1 viruses recovered during the 6^th ^and 7^th ^waves of AI outbreaks in Thailand. The 2008 H5N1 viruses were compared with H5N1 isolates obtained from each wave of AI outbreaks in Thailand. The whole genome sequences of the viruses were analyzed for nucleotide identity, genetic relatedness, virulence determinants, and possible sites of reassortment among H5N1 viruses.

Eight H5N1 viruses were isolated from Nakhon Sawan (n = 3), Phichit (n = 1), Sukhothai (n = 2) and Uthai Thani (n = 2) (Table 1 and Fig 1). The viruses were isolated by embryonated egg inoculation [7]. All 8 viruses were confirmed as Influenza A virus subtype H5N1 by real-time-RT-PCR [8]. Whole genome sequences were obtained as previously described [9]. Phylogenetic and genetic relatedness analyses were conducted using the MEGA 4.0 program applying the neighbor-joining (NJ) approach. Bootstrapping support for tree topologies was also performed with 1000 replicates using MEGA 4.0 [10]. In addition, Bayesian trees were generated using MrBayes V.3.1.2 [11]. Nucleotide identities and genetic analyses were performed using the MegAlign program (DNASTAR). Genetic recombination analysis of H5N1 viruses was performed by Bootscan analysis [12]. Potential sites of genetic reassortment were examined by using tMRCA analysis in a Bayesian Markov chain Monte Carlo BMCMC [13] framework using BEAST version 1.5.4 [11]. Substitution model SRD06 was used. BMCMC runs contained 20 million generations and the convergence of the runs was inspected using Tracer version 1.5. LogCombiner V1.5.4 and Tree Annotator V.1.5.4 were used to combine the results of multiple runs and calculate the mean evolutionary rates and divergence times. Dated phylogeny trees were visualized with Figtree V.1.2.3 [11]. All 8 H5N1 viruses including location and date of isolation as well as nucleotide sequence GenBank accession numbers are shown in table 1.

**Table 1:** List of 2008 H5N1 isolates characterized and analyzed in this study.

Virus	Date of isolation	Location	GenBank accession number
A/Chicken/Thailand/NS-339/08	January, 2008	Nakhon Sawan	EU620652-EU620659
A/Chicken/Thailand/NS-341/08	January, 2008	Nakhon Sawan	EU850413-EU850420
A/Chicken/Thailand/NS-342/08	January, 2008	Nakhon Sawan	EU850421-EU850428
A/Chicken/Thailand/PC-340/08	January, 2008	Phichit	EU620660-EU620667
A/Chicken/Thailand/ST- 351/08	November, 2008	Sukhothai	FJ868014-FJ868021
A/Chicken/Thailand/NIAH-114843/08	November, 2008	Sukhothai	FJ868022-FJ868029
A/Chicken/Thailand/NIAH-115067/08	November, 2008	Uthai Thani	FJ868030-FJ868037
A/Chicken/Thailand/CU-354/08	November 2008	Uthai Thani	CY047456-CY047461

Nucleotide identities of the eight genes showed that all 2008 viruses displayed genetic drift characteristics commonly found in influenza A viruses. The viruses are closely related to clade1 2004-2007 viruses with high nucleotide identity. The percentage of nucleotide identity between 2008 H5N1 viruses (NS-339) and 2004-2008 H5N1 isolates are within the range of 97.4-99.9%(PB2), 97.7-100%(PB1), 97.9-99.9%(PA), 97.8-99.6%(HA), 98.8-99.9%(NP), 98.0-100%(NA), 98.2-99.9%(M) and 98.3-100%(NS) (Table 2). The genetic distance among H5N1 viruses circulating in Thailand over the 5-year course has remained below 3% indicating genetic drift characteristics of the viruses.

**Table 2:** Comparison of the gene segments of A/chicken/NS-339/08 to those of H5N1 viruses from Thailand.

Virus	Nucleotide identity (%)							
	
	**Gene **^**a**^							
	
	PB2	PB1	PA	HA	NP	NA	M	NS
	(73-2220)	(49-2238)	(28-2142)	(16-1667)	(1-1458)	(25-1299)	(1-951)	(10-822)
A/Goose/Guangdong/1/96	92.7	92.5	92.5	95.9	92.9	90.1	95.7	69.2
A/Chicken/Thailand/CU-K2/04	98.0	98.3	98.5	99.0	99.4	98.1	98.8	98.5
A/Chicken/Thailand/CU-23/04	97.8	98.2	98.5	99.0	99.3	98.0	98.7	98.9
A/Chicken/Thailand/NIAH7540/04	97.4	98.5	98.4	98.7	99.1	98.6	98.7	99.0
A/duck/Thailand/NIAH8246/04	98.3	98.6	98.3	98.6	98.9	98.5	98.6	98.8
A/Chicken/Thailand/CK-160/05	98.4	98.7	98.2	98.3	99.0	98.6	98.7	99.4
A/Chicken/Thailand/PC-168/06	97.4	97.7	98.9	99.0	99.7	98.0 ^d^	99.1	98.3
A/Chicken/Thailand/PC-170/06	98.9	99.0	97.9	97.8	98.8	99.1	98.6	99.8
A/Duck/Thailand/CU-329/07	98.1	98.3	97.9	97.9	98.8	98.0	98.2	98.6
A/Chicken/Thailand/ICRC-195/07	99.2 ^b^	99.1	99.2	99.4	99.4 ^c^	99.5	99.4	99.8
A/Chicken/Thailand/ICRC-213/07	98.0	99.1	98.6	98.9	99.3	99.5	98.8	98.8
A/Chicken/Thailand/NS-339/08*	100	100	100	100	100	100	100	100
A/Chicken/Thailand/PC-340/08*	99.9	100	99.9	99.6	99.7	100	99.9	100
A/Chicken/Thailand/NS-341/08*	99.9	99.9	99.8	99.5	99.9	99.8	99.6 ^e^	100
A/Chicken/Thailand/NS-342/08*	99.9	99.9	99.7	99.5	99.9	99.9	99.9	100
A/Chicken/Thailand/ST- 351/08*	98.2	98.4	98.6	98.8	99.5	98.0	98.9	99.0
A/Chicken/Thailand/CU-354/08*	N/A	98.0	N/A	98.6	99.2	99.8	98.5	98.5
A/Chicken/Thailand/NIAH115067/08	99.5	99.7	99.4	99.6	99.7	99.8	99.7	99.4
A/Chicken/Thailand/NIAH114843/08	98.9	99.2	99.1	99.2	99.5	98.9	99.4	99.6

^a ^Region of comparison: position of nucleotides is based on A/chicken/Thailand/NS-339/08 (H5N1)

^b ^Region of PB2 comparison between NS-339 and ICRC-195 at positions 1135-2220

^c ^Region of NP comparison between NS-339 and ICRC-195 at positions 31-687

^d ^Region of NA comparison between NS-339 and PC-168 at positions 25-1209

^e ^Region of M comparison between NS-339 and NS-341 at positions 1-936

**Figure 2:**
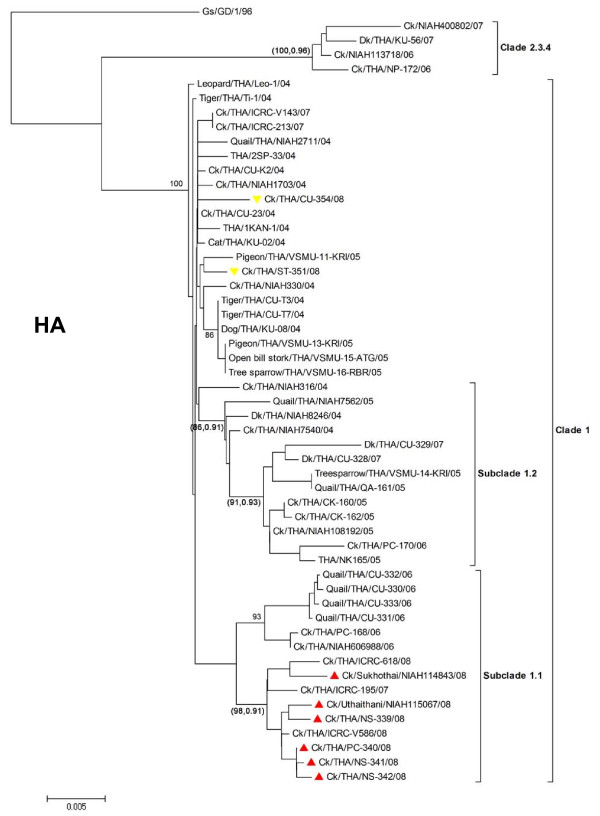
**Phylogenetic trees of HA genes of Thai H5N1 isolates**. The trees were generated using MEGA 4.0 applying the neighbor-joining algorithm. Tree topology was supported by bootstrap analysis with 1000 replicates and posterior probability from BMCMC analysis. The values are shown in parenthesis (NJ/BMCMC). The reassorted 2008 H5N1 viruses are depicted as triangle.

**Figure 3:**
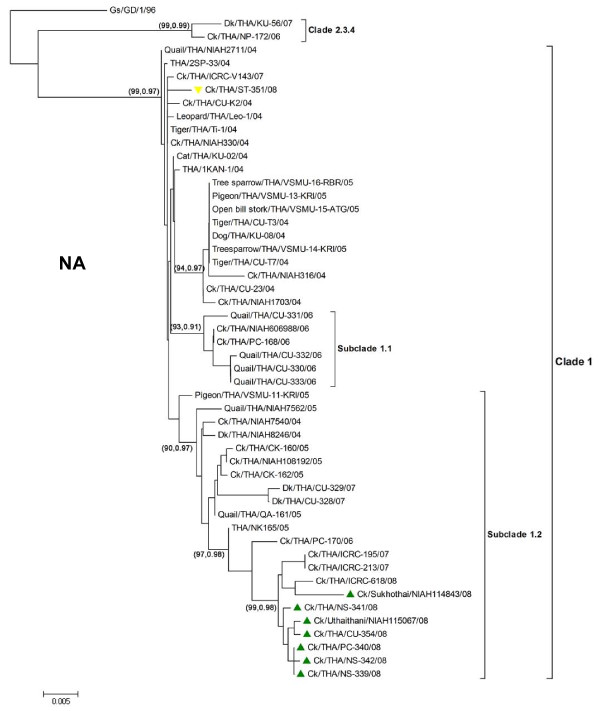
**Phylogenetic trees of NA genes of Thai H5N1 isolates**. The trees were generated using MEGA 4.0 applying the neighbor-joining algorithm. Tree topology was supported by bootstrap analysis with 1000 replicates and posterior probability from BMCMC analysis. The values are shown in parenthesis (NJ/BMCMC). The reassorted 2008 H5N1 viruses are depicted as triangle.

The 2008 H5N1 viruses belong to clade1 which can be further divided into 3 distinct branches including subclade1 (CUK2-like), subclade1.1 (PC168-like) and subclade1.2 (PC170-like) (Fig 2 and Fig 3 and Table 3). Similar findings have been reported previously [3,5]. Phylogenetic analysis of the HA gene showed that six 2008 H5N1 isolates grouped in subclade1.1. On the other hand, two 2008 H5N1 isolates (ST-351 and CU-354) clustered in the original clade1. Unlike the HA gene, phylogenetic analysis of the NA gene showed that seven 2008 H5N1 isolates grouped in another subclade1.2. Phylogenetic analyses of the internal genes of 2008 H5N1 also yielded results similar to those of the HA (PA,NP,M) and NA gene (PB2,PB1,NS). Phylogenetic analysis has shown that the 2008 H5N1 isolates *(n = 6) *are new reassorted H5N1 viruses of subclade1.1 (PA,HA,NP,M) and 1.2 (PB2,PB1,NA,NS). These data suggest that the 2008 H5N1 viruses are either reassortant strains descending from inter-lineage reassortment of subclade1.1 and 1.2 viruses or possibly the original strains circulating since 2004. Similar conclusion has been documented in previous publications [3,6]. Geographically, the reassorted strains were predominantly found in lower-north Thailand, while the original CUK2-like strains mainly circulated in central Thailand.

**Table 3:** Lineage identification of each gene of H5N1 viruses.

Virus	Year	Wave	**Lineage identification **^a^							
			
			PB2	PB1	PA	HA	NP	NA	M**	NS
A/Chicken/Thailand/CU-K2/04	2004	1	1	1	1	1	1	1	1	1
A/Chicken/Thailand/CU-23/04	2004	2	1	1	1	1	1	1	1	1
A/Chicken/Thailand/NIAH7540/04	2004	2	1.1	1	1	1.2	1.2	1.2	1	1
A/duck/Thailand/NIAH8246/04	2004	2	1.2	1	1	1.2	1.2	1.2	1	1
A/Chicken/Thailand/CK-160/05	2005	3	1.2	1.2	1.2	1.2	1.2	1.2	1	1.2
A/Chicken/Thailand/PC-168/06	2006	4	1.1	1.1	1.1	1.1	1.1	1.1	1.1	1.1
A/Chicken/Thailand/PC-170/06	2006	4	1.2	1.2	1.2	1.2	1.2	1.2	1	1.2
A/Duck/Thailand/CU-329/07	2007	5	1.2	1.2	1.2	1.2	1.2	1.2	1	1.2
A/Chicken/Thailand/ICRC-195/07	2007	5	1.2	1.2	1.1	1.1	1.1	1.2	1.1	1.2
A/Chicken/Thailand/ICRC-213/07	2007	5	1	1.2	1	1	1	1.2	1	1
A/Chicken/Thailand/NS-339/08 *	2008	6	1.2	1.2	1.1	1.1	1.1	1.2	1.1	1.2
A/Chicken/Thailand/PC-340/08 *	2008	6	1.2	1.2	1.1	1.1	1.1	1.2	1.1	1.2
A/Chicken/Thailand/NS-341/08*	2008	6	1.2	1.2	1.1	1.1	1.1	1.2	1.1	1.2
A/Chicken/Thailand/NS-342/08*	2008	6	1.2	1.2	1.1	1.1	1.1	1.2	1.1	1.2
A/Chicken/Thailand/ST- 351/08*	2008	7	1	1	1	1	1	1	1	1
A/Chicken/Thailand/CU-354/08*	2008	7	N/A	1	N/A	1	1	1.2	1	1
A/Chicken/Thailand/NIAH115067/08	2008	7	1.2	1.2	1.1	1.1	1.1	1.2	1.1	1.2
A/Chicken/Thailand/NIAH114843/08	2008	7	1.2	1.2	1.1	1.1	1.1	1.2	1.1	1.2

^a ^Lineage identification is based on the topology of Neighbor joining tree with 1000 replication bootstrapping and Bayesian tree with posterior probability of BMCMC analysis

* The viruses characterized in this study.

** M gene clusters in only 2 lineages (lineages 1 and 1.1)

**Figure 4:**
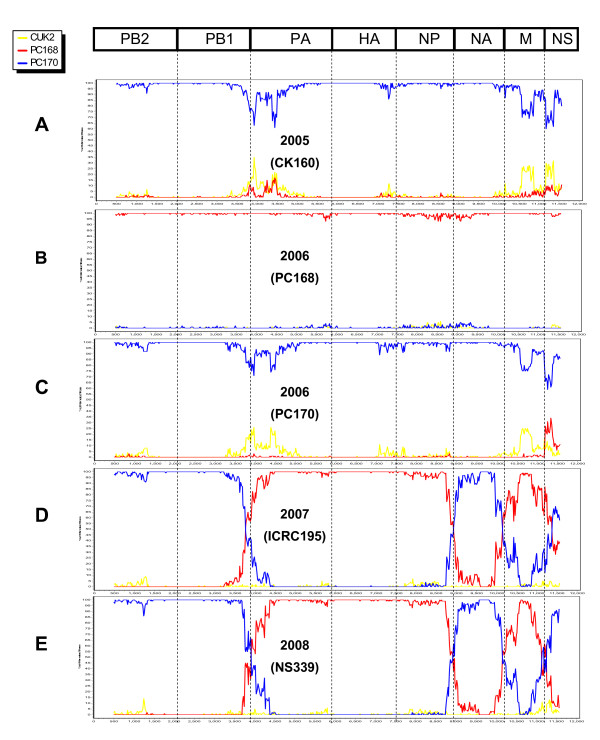
**Bootscan analysis of concatenated H5N1 whole genome sequences**. (A) CK160, (B) PC168, (C) PC170, (D) ICRC195 (E) Reassorted 2008 H5N1 virus (NS339) was used as query sequences. Bootscan analysis was used for the analysis with a window size of 1,000 bp and step size of 20 bp. The red, blue, and yellow lines represent consensus sequences of group 1.1 (2006 H5N1), group 1.2 (2005 and 2006 H5N1), and sequences of early 2004 H5N1 virus.

In order to study the reassortment of the viruses, lineage identification and the time of the most recent common ancestor (tMRCA) of H5N1 isolates representing 7 waves of AI outbreaks were examined. Our result showed that the 2004 H5N1 isolates (NIAH7540 and NIAH 8246) had undergone genetic drift and resulted in a new lineage of PB2, HA, NP and NA genes (Table 3). The tMRCA estimate for the HA and NA genes of NIAH7540 and NIAH8246 was dated to 2003.75 (95%HPD; 2003.41-2004.00) and 2003.65 (95%HPD; 2003.25-2003.97), respectively (Additional files 1, 2 and 3). In 2005-2006, two complete subclades were found as subclade1.1 (PC168) and subclade1.2 (CK160 and PC170). The tMRCA estimate for HA gene of PC168 and PC170 was dated to 2005.79 and 2004.78, respectively. This result suggests that the new subclades 1.1 and 1.2 of H5N1 in Thailand originated between 2004 and 2005. From 2007 to 2008, one 2007 H5N1 isolate (ICRC195) and 6 2008 H5N1 isolates (NS-339, PC-340, NS-341, NS342, NIAH-114843 and NIAH-115067) became new reassorted viruses between subclades1.1 and 1.2 (Table 3). The tMRCA estimate for 2007-2008 reassorted viruses was dated to 2006.25-2007.82 (95%HPD; 2005.45-2008.00) (Additional files1,2 and 3). This result suggests that the potential site of genetic reassortment of Thai H5N1 originated between 2006 and 2007. The new reassorted viruses circulating in Thailand since 2007 may be the result of a strong bottleneck effect that caused only a few lineages to remain in the area resulting in genetic reassortment [6]. However, original clade H5N1 viruses (ST-351 and CU-354) still circulated. To confirm reassortment among H5N1 isolates, the viruses were analyzed using Bootscan analysis [12,14]. The Bootscan plots of 2005-2006 H5N1 (CK160 and PC170) show high sequence identity to subclade1.2, while genes of 2006 H5N1 (PC168) have high sequence similarity to subclade 1.1 (Fig 4). Bootscan analysis of 2007 H5N1 (ICRC-195) and 2008 H5N1 (NS-339,PC-340,NS-341,NS-342) shows high sequence identity between the putative reassorted viruses and group1.1 in the PA,HA,NP,M genes, and group 1.2 in the PB2,PB1,NA,NS genes. Bootscan plot supports the recombinant points near the junctions of PB1/PA, NP/NA, NA/M and M/NS in the concatenated genomes of 2008 H5N1 viruses (Fig 4). This result confirms that the new reassorted viruses had acquired four gene segments from group1.1 and other genes from group1.2 [3]. It is noted that Bootscan and tMRCA analysis have been used to confirm reassortment of H5N1viruses from Indonesia [14].

The 2008 H5N1 isolates displayed HPAI characteristics similar to those of the 2004-2007 (genotype Z) viruses, but different from genotype V viruses [1,4] (Table 4). The HA gene contained the polybasic amino acids at the HA cleavage site indicating HPAI characteristics [15]. Amino acids related to the receptor binding site Q222-G224 have remained unchanged indicating the viruses preferentially bind to the avian receptor [16]. All 2008 viruses contained 7 N-link glycosylation sites which have remained unaltered since 2004. Conservation of the 154-156 glycosylation site in Thai isolates but not in the clade2.2 viruses might indicate the virulence factors of the virus in the region [17]. All 2008 H5N1 have retained amino acid residues at positions 83(A), 86(V) and 141(S) similar to 2004-2007 H5N1 viruses. However, some 2008 H5N1 isolates (except for ST-351 and CU-354) display amino acid polymorphisms at the antigenic site E at positions 138(Q138L) and 140(K140R). The results indicate that 2008 H5N1 viruses had undergone genetic drift and thus, amino acid changes at residues 138 and 140. As for NA, the 2008 H5N1 viruses display 20 amino acid deletions in the NA stalk region indicating evolutionary changes of the viruses [16]. The 2008 H5N1 viruses do not harbor any mutation at amino acid positions E119,H275,R293 and N295 of NA proven responsible for Oseltamivir resistance which fortunately suggests Oseltamivir sensitive viruses [18-21]. On the other hand, the 2008 viruses show Amantadine resistant amino acids in M2 at L26I and S31N but not at positions 27,30,64,66 [22-24]. In NS1, the 2008 H5N1 viruses contain 5 amino acid deletions which have remained unchanged since 2004. As for NS1, D92 at position 92 has remained unaltered which is related to virulence in mammalian species due to the antiviral effects of interferons and TNF [25]. The 2008 H5N1 viruses contain ESEV residues at the C-terminus of NS1 indicating high virulence [26]. As for the PB2 gene, the viruses contain E627 indicating low virulence in mammalian species [27]. An E627K mutation in PB2 would indicate increased viral replication efficiency in mammals [28]. Analysis of amino acids associated with human and avian H5N1 viruses indicated that the reassorted 2008 H5N1 viruses possess both human and avian characteristics. It is noted that the virulence of these reassortant viruses will need to be further confirmed in an animal model to validate their pathogenicity.

**Table 4:** Genetic analysis of the amino acid sequences of the proteins of 2004-2008 Thai H5N1 isolates.

	HA														NA			
	
Virus	HA Cleavage site			Receptor binding site		N-link Glycosylation site	Antigenic site E		Antigenic site A			NA stalk region			Oseltamivir resistant amino acid			
	
	320-331			222	224	154-156	83	86	138	140	141	49-68			119	275	293	295
A/Goose/Guangdong/1/96	TPQRERRRKKR/G			Q	G	(NSA)	A	A	H	R	S	No deletion			E	H	R	N
A/Chicken/Thailand/CU-K2/04	SPQRERRRKKR/G			Q	G	NST	A	V	Q	K	S	20 aa deletion			E	H	R	N
A/Chicken/Thailand/CU-23/04	SPQRERRRKKR/G			Q	G	NST	A	V	Q	K	S	20 aa deletion			E	H	R	N
A/Chicken/Thailand/NIAH7540/04	SPQREKRRKKR/G			Q	G	NST	A	V	Q	K	S	20 aa deletion			E	H	R	N
A/duck/Thailand/NIAH8246/04	SPQREKRRKKR/G			Q	G	NST	A	V	Q	K	S	20 aa deletion			E	H	R	N
A/Chicken/Thailand/CK-160/05	SPQREKRRKKR/G			Q	G	NST	A	A	Q	K	S	20 aa deletion			E	H	R	N
A/Chicken/Thailand/PC-168/06	SPQRERRRKKR/G			Q	G	NST	A	V	L	K	S	20 aa deletion			E	H	R	N
A/Chicken/Thailand/PC-170/06	SPQREKRRKKR/G			Q	G	NST	P	A	Q	K	S	20 aa deletion			E	H	R	N
A/Duck/Thailand/CU-329/07	SPQREKRRKKR/G			Q	G	NST	A	A	L	N	P	20 aa deletion			E	H	R	N
A/Chicken/Thailand/ICRC-195/07	SPQRERRRKKR/G			Q	G	NST	A	V	L	R	S	20 aa deletion			E	H	R	N
A/Chicken/Thailand/ICRC-213/07	SPQRERRRKKR/G			Q	G	NST	A	V	Q	K	S	20 aa deletion			E	H	R	N
A/Chicken/Thailand/NS-339/08*	SPQRERRRKKR/G			Q	G	NST	A	V	L	R	S	20 aa deletion			E	H	R	N
A/Chicken/Thailand/PC-340/08*	SPQRERRRKKR/G			Q	G	NST	A	V	L	R	S	20 aa deletion			E	H	R	N
A/Chicken/Thailand/NS-341/08*	SPQRERRRKKR/G			Q	G	NST	A	V	L	R	S	20 aa deletion			E	H	R	N
A/Chicken/Thailand/NS-342/08*	SPQRERRRKKR/G			Q	G	NST	A	V	L	R	S	20 aa deletion			E	H	R	N
A/Chicken/Thailand/ST- 351/08*	SPQRERRRKKR/G			Q	G	NST	A	V	Q	K	S	20 aa deletion			E	H	R	N
A/Chicken/Thailand/CU-354/08*	SPQRERRRKKR/G			Q	G	NST	A	V	Q	K	S	20 aa deletion			E	H	R	N
A/Chicken/Thailand/NIAH115067/08	SPQRERRRKKR/G			Q	G	NST	A	V	L	R	S	20 aa deletion			E	H	R	N
A/Chicken/Thailand/NIAH114843/08	NPQRERRRKKR/G			Q	G	NST	A	V	L	R	S	20 aa deletion			E	H	R	N

**Virus**	**Gene**																	
	
	**M2**									**NS1**			**PB2**			**PB1**	**PA**	**NP**
	
	**Amantadine-resistant amino acids**						**Human/Avian-like characteristics**			**5-aa deletion**	**Virulence determinant**		**Virulence determinant**			**Virulence**	**H/A**	**H/A**
	
	**26**	**27**	**30**	**31**	**64**	**66**	**16**	**28**	**55**	**80-84**	**92**	**c-terminal**	**627**	**661**	**702**	**198**	**409**	**136**
	
A/Goose/Guangdong/1/96	L	V	A	S	S	E	E	V	L	No	D	ESEV	E	A	K	K	S	M
A/Chicken/Thailand/CU-K2/04	I	V	A	N	A	A	E	V	L	Yes	D	ESEV	E	A	K	K	S	L
A/Chicken/Thailand/CU-23/04	I	V	A	N	A	A	E	V	L	Yes	N	ESEV	E	A	K	K	S	L
A/Chicken/Thailand/NIAH7540/04	I	V	A	N	A	A	E	V	L	Yes	D	ESEV	E	A	K	K	S	L
A/duck/Thailand/NIAH8246/04	I	V	A	N	A	A	E	V	L	Yes	D	ESEV	E	A	K	K	S	L
A/Chicken/Thailand/CK-160/05	I	V	A	N	A	A	E	V	L	Yes	D	ESEV	E	A	K	K	S	L
A/Chicken/Thailand/PC-168/06	I	V	A	N	A	A	E	V	L	Yes	D	ESEV	E	A	K	K	S	L
A/Chicken/Thailand/PC-170/06	I	V	A	N	A	A	E	V	L	Yes	D	ESEV	E	A	K	K	S	L
A/Duck/Thailand/CU-329/07	I	V	A	N	A	A	E	V	L	Yes	D	ESEV	E	A	K	K	S	L
A/Chicken/Thailand/ICRC-195/07	I	V	A	N	A	A	E	V	L	Yes	D	ESEV	E	A	K	K	S	L
A/Chicken/Thailand/ICRC-213/07	I	V	A	N	A	A	E	V	L	Yes	D	ESEF	E	A	K	K	S	L
A/Chicken/Thailand/NS-339/08*	I	V	A	N	A	A	E	V	F	Yes	D	ESEV	E	A	K	K	S	L
A/Chicken/Thailand/PC-340/08*	I	V	A	N	A	A	E	V	F	Yes	D	ESEV	E	A	K	K	S	L
A/Chicken/Thailand/NS-341/08*	I	V	A	N	A	A	E	V	F	Yes	D	ESEV	E	A	K	K	S	L
A/Chicken/Thailand/NS-342/08*	I	V	A	N	A	A	E	V	F	Yes	D	ESEV	E	A	K	K	S	L
A/Chicken/Thailand/ST- 351/08*	I	V	A	N	A	A	E	V	L	Yes	D	ESEV	E	A	K	K	S	L
A/Chicken/Thailand/CU-354/08*	I	V	A	N	A	A	E	V	L	Yes	D	ESEV	-	-	-	K	-	L
A/Chicken/Thailand/NIAH115067/08*	I	V	A	N	A	A	E	V	F	Yes	D	ESEV	E	A	K	K	S	L
A/Chicken/Thailand/NIAH114843/08*	I	V	A	N	A	A	E	V	L	Yes	D	ESEV	E	A	K	K	S	L

In conclusion, the clade nomenclature of H5N1 viruses has been determined based on the HA gene [29]. The viruses examined in this study have undergone inter-lineage reassortment and thus represent the genetic parents of the new reassorted 2008 H5N1 viruses. The potential site of genetic reassortment of Thai 2008 H5N1 could be traced backed to 2006. Bootscan analysis of the 2008 H5N1 viruses supported the reassortment sites. The new reassorted viruses have acquired four gene segments (PA,HA,NP,M) from subclade1.1 and four genes (PB2,PB1,NA,NS) from subclade1.2. This finding raises concerns with regard to circulation of newly reassorted viruses in Thailand. Geographically, the 2008 reassorted lineages predominantly circulated in the lower-north provinces of Thailand, while the original CUK2 lineage is mainly found in the central provinces. This phenomenon may be due to the strong bottle-neck effect in the region especially in the lower north of Thailand. To prevent future reassortment, emphasis should be put on prevention and control of AI outbreaks in the lower north of Thailand, not only in avian species but also in mammals [30,31], since a human-animal interface could create any emerging virus similar to the recently emerged pandemic H1N1 [32]. In addition, surveillance and whole genome sequencing of H5N1 viruses should be routinely performed for monitoring the genetic drift of the virus and identifying new reassorted strains, especially in light of potential reassortment between avian and mammalian H5N1 viruses.

## Competing interests

The authors declare that they have no competing interests.

## Authors' contributions

AA designed H5N1 surveillance, data analyses and final approval of the manuscript. JL, KS, MW, and TW conducted genome sequencing and phylogenetic analysis. PK, SS, AT, YP, JS and RT performed H5N1 outbreak investigation and drafted the manuscript. SB and NB, RT participated in sample collection, virus isolation and whole genome sequencing. All authors read and approved the final manuscript.

## Supplementary Material

Additional file 1 **Estimated time of the most recent common ancestor (tMRCAs) for Thai H5N1 viruses**. Table of the estimated time of the most recent common ancestor (tMRCAs) for Thai H5N1 viruses. Click here for file

Additional file 2 **Dated phylogenetic tree of the HA of Thai H5N1 viruses**. Dated phylogenetic tree of the HA of Thai H5N1 viruses. The tree is scaled to time (1996-2008) and was generated using the SRD06 codon model and uncorrelated relaxed clock model. The top panel shows average tMRCAs and 95% HPDs of tMRCAs for H5N1 viruses in the study. Click here for file

Additional file 3 **Dated phylogenetic tree of the NA of Thai H5N1 viruses**. Dated phylogenetic tree of the NA of Thai H5N1 viruses. The tree is scaled to time (1996-2008) and was generated using the SRD06 codon model and uncorrelated relaxed clock model. The top panel shows average tMRCAs and 95% HPDs of tMRCAs for H5N1 viruses in the study. Click here for file
