# Supplementary material for: Genetic characterization of 2008 reassortant influenza A virus (H5N1), Thailand
Source: Virol J. 2010 Sep 16;7:233. doi: 10.1186/1743-422X-7-233 (PMC2949837; doi:10.1186/1743-422X-7-233)
Supplement: Additional file 1 — Estimated time of the most recent common ancestor (tMRCAs) for Thai H5N1 viruses. Table of the estimated time of the most recent common ancestor (tMRCAs) for Thai H5N1 viruses. [file 1743-422X-7-233-S1.DOC]

HA gene

| Virus strains | Year | Wave | Lineage | Mean TMRCA (95% HPD) |
| --- | --- | --- | --- | --- |
|  |  |  |  |  |
| A/Chicken/Thailand/CU-K2/04 | 2004 | 1 | 1 | 2003.68 (2003.38-2003.96) |
| A/Chicken/Thailand/CU-23/04 | 2004 | 2 | 1 | 2003.78 (2003.47-2004.00) |
| A/Chicken/Thailand/NIAH7540/04 | 2004 | 2 | 1.2 | 2003.75 (2003.41-2004.00) |
| A/duck/Thailand/NIAH8246/04 | 2004 | 2 | 1.2 | 2003.65 (2003.25-2003.97) |
| A/Chicken/Thailand/CK-160/05 | 2005 | 3 | 1.2 | 2004.85 (2004.59-2005.00) |
| A/Chicken/Thailand/PC-168/06 | 2006 | 4 | 1.1 | 2005.79 (2005.45-2006.00) |
| A/Chicken/Thailand/PC-170/06 | 2006 | 4 | 1.2 | 2004.78 (2004.50-2004.99) |
| A/Duck/Thailand/CU-329/07 | 2007 | 5 | 1.2 | 2006.04 (2005.17-2006.78) |
| A/Chicken/Thailand/ICRC-195/07 | 2007 | 5 | 1.1 | 2006.25 (2005.45-2006.90) |
| A/Chicken/Thailand/ICRC-213/07 | 2007 | 5 | 1 | 2006.90 (2006.63-2007.00) |
| A/Chicken/Thailand/NS-339/08 | 2008 | 6 | 1.1 | 2007.56 (2007.12-2007.92) |
| A/Chicken/Thailand/PC-340/08 | 2008 | 6 | 1.1 | 2007.82 (2007.51-2008.00) |
| A/Chicken/Thailand/NS-341/08 | 2008 | 6 | 1.1 | 2007.82 (2007.51-2008.00) |
| A/Chicken/Thailand/NS-342/08 | 2008 | 6 | 1.1 | 2007.64 (2007.21-2007.96) |
| A/Chicken/Thailand/ST-351/08 | 2008 | 7 | 1 | 2007.04 (2006.26-2007.81) |
| A/Chicken/Thailand/NIAH-114843/08 | 2008 | 7 | 1.1 | 2007.27 (2006.61-2007.84) |
| A/Chicken/Thailand/NIAH-115067/08 | 2008 | 7 | 1.1 | 2007.56 (2007.12-2007.92) |
| A/Chicken/Thailand/CU-354/08 | 2008 | 7 | 1 | 2007.04 (2006.26-2007.81) |
|  |  |  |  |  |

**NA gene**

| Virus strains | Year | Wave | Lineage | Mean TMRCA (95% HPD) |
| --- | --- | --- | --- | --- |
|  |  |  |  |  |
| A/Chicken/Thailand/CU-K2/04 | 2004 | 1 | 1 | 2003.68 (2003.38-2003.96) |
| A/Chicken/Thailand/CU-23/04 | 2004 | 2 | 1 | 2003.78 (2003.47-2004.00) |
| A/Chicken/Thailand/NIAH7540/04 | 2004 | 2 | 1.2 | 2003.77 (2003.44-2004.00) |
| A/duck/Thailand/NIAH8246/04 | 2004 | 2 | 1.2 | 2003.55 (2003.12-2003.89) |
| A/Chicken/Thailand/CK-160/05 | 2005 | 3 | 1.2 | 2004.85 (2004.59-2005.00) |
| A/Chicken/Thailand/PC-168/06 | 2006 | 4 | 1.1 | 2005.79 (2005.45-2006.00) |
| A/Chicken/Thailand/PC-170/06 | 2006 | 4 | 1.2 | 2004.78 (2004.50-2004.99) |
| A/Duck/Thailand/CU-329/07 | 2007 | 5 | 1.2 | 2006.04 (2005.17-2006.78) |
| A/Chicken/Thailand/ICRC-195/07 | 2007 | 5 | 1.2 | 2006.25 (2005.45-2006.90) |
| A/Chicken/Thailand/ICRC-213/07 | 2007 | 5 | 1.2 | 2006.90 (2006.63-2007.00) |
| A/Chicken/Thailand/NS-339/08 | 2008 | 6 | 1.2 | 2007.57 (2007.12-2007.93) |
| A/Chicken/Thailand/PC-340/08 | 2008 | 6 | 1.2 | 2007.83 (2007.54-2008.00) |
| A/Chicken/Thailand/NS-341/08 | 2008 | 6 | 1.2 | 2007.83 (2007.54-2008.00) |
| A/Chicken/Thailand/NS-342/08 | 2008 | 6 | 1.2 | 2007.65 (2007.23-2007.95) |
| A/Chicken/Thailand/ST-351/08 | 2008 | 7 | 1 | 2007.05 (2006.20-2007.78) |
| A/Chicken/Thailand/NIAH-114843/08 | 2008 | 7 | 1.2 | 2007.29 (2006.64-2007.84) |
| A/Chicken/Thailand/NIAH-115067/08 | 2008 | 7 | 1.2 | 2007.57 (2007.12-2007.93) |
| A/Chicken/Thailand/CU-354/08 | 2008 | 7 | 1.2 | 2007.05 (2006.20-2007.78) |
|  |  |  |  |  |
