# Supplementary figures and images for: Genetic characterization of 2008 reassortant influenza A virus (H5N1), Thailand
Source: Virol J. 2010 Sep 16;7:233. doi: 10.1186/1743-422X-7-233 (PMC2949837; doi:10.1186/1743-422X-7-233)

## Slide 1
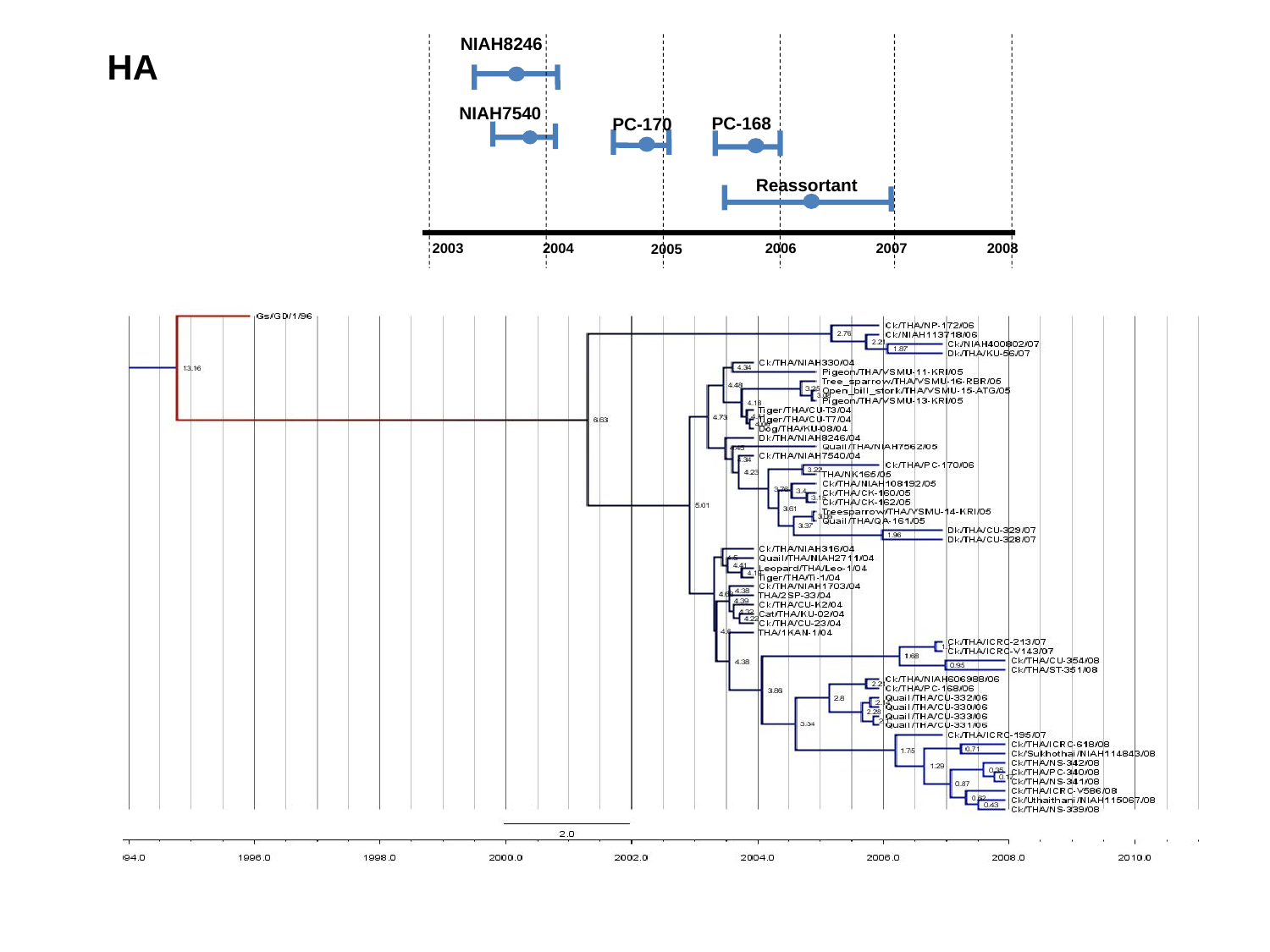

NIAH8246
HA
NIAH7540
PC-170
PC-168
Reassortant
2003
2004
2006
2007
2008
2005

Supplement: Additional file 2 — Dated phylogenetic tree of the HA of Thai H5N1 viruses. Dated phylogenetic tree of the HA of Thai H5N1 viruses. The tree is scaled to time (1996-2008) and was generated using the SRD06 codon model and uncorrelated relaxed clock model. The top panel shows average tMRCAs and 95% HPDs of tMRCAs for H5N1 viruses in the study. [file 1743-422X-7-233-S2.PPT]

## Slide 1
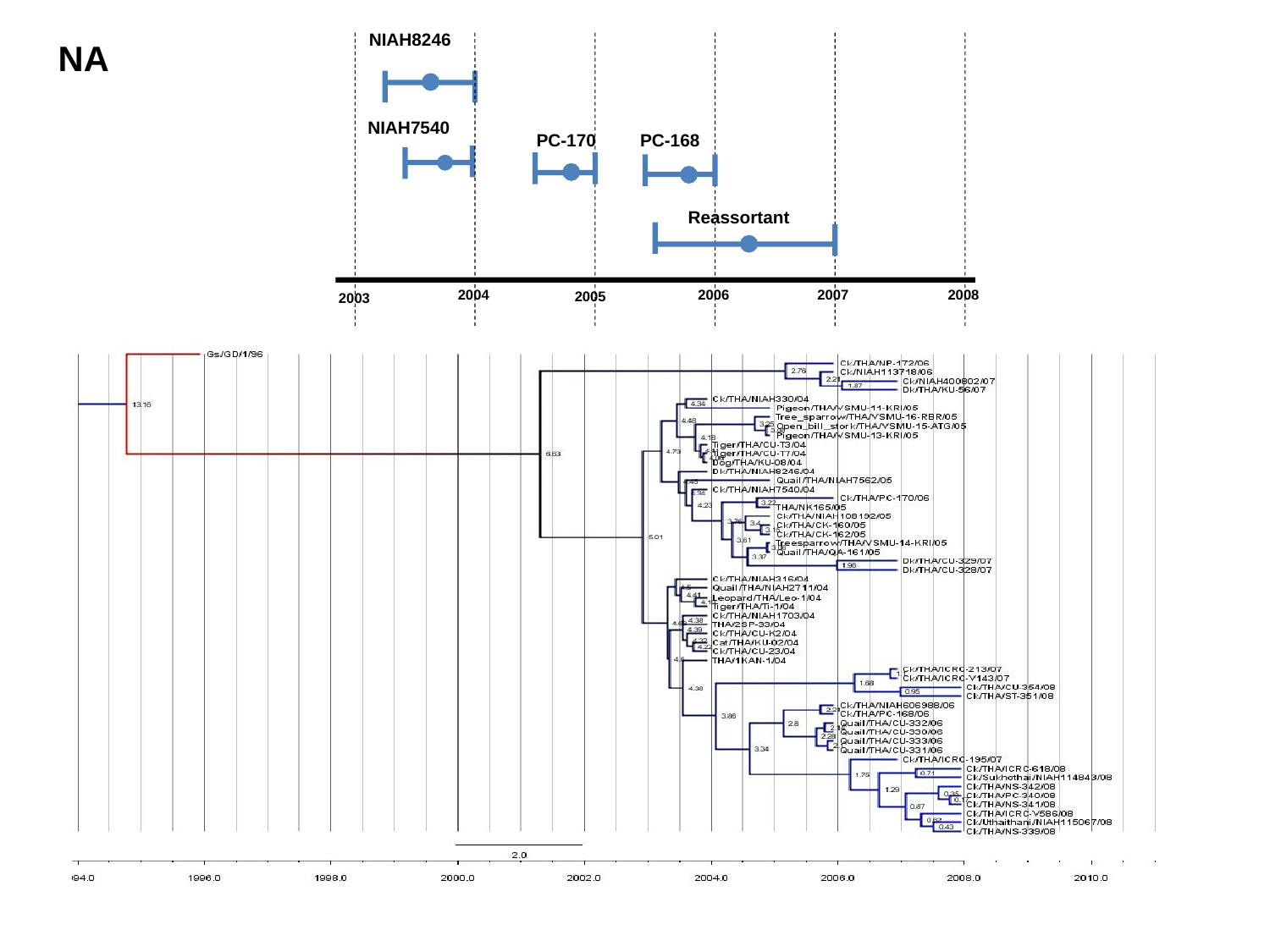

NIAH8246
NA
NIAH7540
PC-170
PC-168
Reassortant
2004
2006
2007
2008
2005
2003

Supplement: Additional file 3 — Dated phylogenetic tree of the NA of Thai H5N1 viruses. Dated phylogenetic tree of the NA of Thai H5N1 viruses. The tree is scaled to time (1996-2008) and was generated using the SRD06 codon model and uncorrelated relaxed clock model. The top panel shows average tMRCAs and 95% HPDs of tMRCAs for H5N1 viruses in the study. [file 1743-422X-7-233-S3.PPT]
